# Disparity between knowledge and practice regarding COVID-19 in Thailand: A cross-sectional study of older adults

**DOI:** 10.1371/journal.pone.0259154

**Published:** 2021-10-26

**Authors:** Paolo Miguel Manalang Vicerra

**Affiliations:** College of Population Studies, Chulalongkorn University, Bangkok, Thailand; Health Services Academy Islamabad Pakistan, PAKISTAN

## Abstract

The efficacy of the public health measures to mitigate COVID-19 is influenced by health literacy which includes the level of knowledge about the disease and the preventive behaviours adopted by individuals. Thailand, being a low- and middle-income country with an ageing society, has to consider both the challenges that its health system has in disseminating information and the disparities in health literacy among its older population. This study investigated the knowledge and behaviour of older adults in Thailand regarding COVID-19 using the Impact of COVID-19 on Older Persons in Thailand, a cross-sectional survey. The data was primarily collected online and included 1,230 adults aged at least 60 years from nine provinces of the five regions of the country. The associated factors with the health literacy outcomes were tested using bivariate logistic regression analyses. It was observed that 43% of the older adults in the sample had proper knowledge of the disease and 33% adopted preventive behaviours. Knowledge about the disease was not associated with preventive behaviour. The associated factors common between the increased levels of knowledge and adoption of behaviours were rural area residence and higher educational attainment levels. Obtaining information from the internet was observed to increase knowledge while having the television and radio as sources of information had negative relationship. Many older adults continued to be employed during the lockdown period and this was associated with decreased adoption of preventive behaviour. The context of vulnerable populations, particularly older adults, is different with regard to their access to information and concern about income. Health information has to be tailored for targeted populations. Their needs also have to be addressed as they have increased risks because of financial and health susceptibilities.

## Introduction

The coronavirus disease (COVID-19) is from the SARS-CoV-2, an airborne virus [1]. Particles in the air that have viral content are able to travel metres from the origin. Previous epidemics such as the severe acute respiratory syndrome (SARS) and the H1N1 influenza were similarly transmitted in the air therefore, indoor environments were particularly risk-laden and proper ventilation has been noted to be a mode of decreasing the rate of transmission [2]. For COVID-19, various measures were implemented across many countries including handwashing, wearing face masks, distancing physically, and sheltering in place [3]. Diagnostic testing was also emphasised in order to place COVID-19-positive individuals in quarantine and perform contact-tracing which can mitigate the transmission.

Adherence to public health measures to control the spread of the virus was impacted by social and environmental contexts. Some have noted that such interventions pose difficulties among low- and middle-income countries (LMICs) [4, 5]. The number of people in a household may be higher in LMIC settings and the transmutability of the virus is increased. It is also imperative for some people to continue working and earn income which carries risks. Another source of gradient is health systems where medical supplies and human resources is in need of strengthening even prior the occurrence of COVID-19 [6]. The procurement of necessary preventive tools such as the personal protective equipment was made difficult across countries because of shortages [7].

In the situation of a pandemic, health promotion is essential [8]. It is necessary for people to have the capacity to obtain, evaluate, and implement health information and develop their health status [9]. Compliance to public health measures then requires understanding and developing a certain level of perceived risks with regard to the situations. An aspect though that had garnered less attention concerns the ability of people to comply as individuals can have restrictions on their financial and social resources [10]. Equitable health measures and promotion need to be emphasised to place the needs of vulnerable populations, including older people, in the foreground. The said challenges to address COVID-19 are compounded in an ageing society with a developing economy such as Thailand.

Thailand’s population structure developed toward being an ageing society in the 1980s when the proportion of people aged at least 60 years increased and fertility levels of the country continuously decreased [11]. In 2020, people in their older ages comprise 19% of the total Thai population [12]. The life expectancy of this age group has been increasing over the years [13]. Most of them continue to engage in gainful employment because it is their sole source of income [14]. The current older population were part of the informal economy in their younger adult years and therefore they are not members of the pension system. In lieu of this, the development of the means of controlling the transmission of COVID-19 in Thailand is notable as international border closure and national lockdown measures were imposed on 26 March 2020. This had affected the social and financial aspects of the people which may have influenced their access to information and behaviour during the pandemic.

This study investigated the health literacy of older persons in Thailand during a pandemic in terms of knowledge and behaviour regarding COVID-19. These are important cognitive facets on public health efforts toward curbing the transmission of the disease. In this present study, the level of knowledge and the adherence to recommended practices for self-protection among the older population were observed as separate but related matters in order to observe if the social characteristics that were associated with either outcome were similar. The points of dissimilarities were considered to be issues that indicate the gap between knowledge and behaviour. Studies on knowledge and practices or behaviour related to COVID-19 had been done for other LMICs as Bangladesh [15] which observed knowledge, attitude and practice among individuals aged 12–64 years; and the Philippines [16] which analysed health literacy among adults including a sub-sample of people in older ages. Other studies on populations of Nigeria [17] and Malaysia [18] have also been done but there is a shortcoming in the literature that focuses on knowledge and behaviour solely among older adults.

## Materials and methods

### Data

The current study utilised the Impact of COVID-19 on Older Persons in Thailand survey conducted in July 2020 by College of Population Studies, Chulalongkorn University [19]. The date for data collection was selected because it was after lockdown measures were abated. A multistage sampling method was employed. Thailand was first stratified into the five including the northern, central, north eastern, and southern regions and Bangkok metropolitan area. Each stratum was categorised to urban and rural areas. Then, two provinces were selected. To ensure a 50% minimum response rate, a criterion for one of the said provinces for each region was selected based on the size of the proportion of its older population. For the other province, the proportion of older people with high economic vulnerability was selected. Further consideration was provided in terms of residence because it was estimated that about 59% of older persons in the country were in the rural areas. As a result, the total of 1,230 persons aged at least 60 years were included the sample.

The survey was primarily designed to be a self-administered online questionnaire which was distributed by local community intermediaries. The local intermediaries were local administrative officers, health staff, or part of the village health volunteers in the communities. An alternative face-to-face data collection method was implemented if the respondent has limited literacy, has poor visual acuity, or does not have a smartphone or any device to connect to the internet. The rationale and objectives of the survey were presented in the online survey. It was then followed by a question regarding the provision of consent by the respondent to confirm their understanding of the aim and use of the survey and to ensure them of confidentiality of information. This was therefore recorded within the dataset as an item response in the online survey. The author was able to utilise completely anonymised data. The ethical approval for the conduct and use of the survey was granted by the Research Ethics Review Committee for Research Involving Human Research Participants, Health Sciences Group of Chulalongkorn University.

### Measures

#### Sociodemographic measures

Social and demographic information was collected including age, gender, marital status (Never married/separated/widowed & married), living arrangement (alone, with spouse only, with at least one child, & with other people i.e. caretaker), education level (none and lower than primary level, primary level [4^th^ to 6^th^ grades], & higher than primary level), employment status (not employed during the outbreak & those employed), and average annual income prior the pandemic (<10,000 Thai Baht. 10,000–29,999 Thai Baht, & ≥30,000 Thai Baht).

#### Sources of information

The measures for this are based on two items in the survey: (1) the ownership or access to appliances or gadgets including radio, television (TV), mobile phone/smartphone, desktop/laptop/tablet, and the internet; and (2), if the respondent obtained COVID-19-related information from those appliances or devices during the lockdown period. Two measures were created based on the abovementioned details: (1) Owns radio/TV and received information through them and (2) Owns mobile phone/computer and obtained information on COVID-19 through SMS or the internet.

#### Knowledge about COVID-19

Respondents were enquired about four statements relating to COVID-19 and they were to respond if they were correct or otherwise. An index was created for this item where all appropriate responses were counted (Cronbach’s α: 0.65). Cronbach’s alpha value is affected by the number of items that comprise the scale and as such, the current scale is at the lower limit of the adequate value which is at the range of 0.65–0.8 [20]. A dichotomous measure was then created where the unity was having the maximum score of four (4) was obtained.

#### Preventive behaviour regarding COVID-19

To assess the range of practices which respondents adopt to protect themselves from the disease, five questions were asked whereby the response options were never, sometimes, and always. An index was created by counting the frequency where ‘always’ was the response (Cronbach’s α: 0.72). A dichotomous measure was created for this variable where having the maximum score of five (5) was the outcome category.

#### Worries during COVID-19 pandemic

This measure is included to provide further description of the situation among the respondents but is not a part of the model analyses. Among the questions in the survey was indicating particular concerns the respondents experienced after being asked if they had any worries or concerns during the COVID-19 outbreak. Seven items were presented to individuals which are: fear of myself or family being infected with COVID-19, having worse health because of missed medical appointments, personal and family financial status, accessibility to treatment for COVID-19, conflict with family while co-residing, living alone if family members were to be infected, and being unable to purchase food and other essentials. Respondents can answer all that were applicable to them. An open-ended item for concerns was included in the survey but none had cited anything different from those that have been mentioned.

### Analytic strategy

Descriptive statistics and bivariate statistics (χ^2^) were done. Urban-rural residence was selected for the bivariate distribution upon considering age groups and gender. Residence was correlated with both knowledge of COVID-19 (χ^2^ = 50.9, *p*-value <0.001) and preventive behaviour (χ^2^ = 5.9, *p*-value = 0.015). Considering age groups yielded a lack of correlation with knowledge (χ^2^ = 1.6, *p*-value = 0.458) but correlated with behaviour (χ^2^ = 10.9, *p*-value = 0.004). For gender, no correlation was observed with either knowledge (χ^2^ = 0.2, *p*-value = 0.632) or preventive practices (χ^2^ = 0.5, *p*-value = 0.446). The bivariate statistics were performed as a means to understand the distribution of characteristics and health literacy of the sample but the subsequent multivariate analyses considered the general sample without the distinction by residence.

For the multivariate analyses, bivariate logistic regression model analyses were done with 95% confidence interval to determine the association of sociodemographic and information sources variables with the outcome variables knowledge and preventive behaviour regarding COVID-19.

## Results

The total sample was comprised mostly of those in the age group of 60 to 69 years old (58%) (Table 1). More than half of the sample were female (55%) and married (64%). Many of the respondents were also living with at least one child (68%). The socioeconomic profile of the older adults in the sample was that a majority had primary level of education attainment (69%) and some had at least 30,000 Baht in annual income (43%). Almost all owned a television and a radio and they had obtained information about COVID-19 from those media sources during the pandemic (95%) while much fewer had mobile devices or computers that were internet-capable (27%). Continuing in Table 1, a comparison of the composition between residences had shown a majority of the sample were from rural areas (58%). A notable characteristics composition difference is that more of the older adults in the rural areas had lower levels of annual income compared with urban residents.

**Table 1:** Characteristic distribution of sample and residential differences.

	Total		Urban (%)	Rural (%)	*p*-value*
	n	%			
**Age groups**					
60–69	707	57.5	47.1	52.9	<0.001
70–79	376	30.6	34.6	65.4	
80+	147	12.0	37.4	62.6	
**Female**	682	55.4	42.5	57.5	0.746
**Married**	784	63.7	41.8	58.2	0.794
**Living arrangement**					
Living alone	68	5.5	48.5	51.5	0.517
Living with spouse only	147	12.0	45.6	54.4	
Living with at least one child	836	68.0	41.0	59.0	
Living with other people	179	14.6	41.9	58.1	
**Education attainment**					
Lower than primary level	91	7.4	29.7	70.3	0.013
Primary level [4–6 years]	845	68.7	41.8	58.2	
Higher than primary level	294	23.9	46.9	53.1	
**Employed during COVID-19 pandemic**	109	8.9	39.5	60.6	0.555
**Average annual income**					
<10,000	347	28.2	33.1	66.9	<0.001
10,000–29,999	353	28.7	38.8	61.2	
≥30,000	530	43.1	50.2	49.8	
**Owns radio/TV and received COVID-19 information through them**	1172	95.3	43.3	57.7	0.509
**Owns mobile phone/computer and obtained information on COVID-19 through SMS or the internet**	331	26.9	47.4	52.6	0.022
**Total**	1230	100	42.1	57.9	

Source: Impact of COVID-19 on Older Persons in Thailand Survey.

*Based on χ^2^ test comparing urban and rural residence.

There were social ramifications due to the COVID-19 pandemic and individual concerns were observed (Table 2). The primary concern of the people overall within the survey sample was the risk of infection (41%). Other leading worries were the financial status and also the missed appointments with health personnel. The order of concerns differed when urban and rural differences were described. Conflict with family while living together and personal and familial financial status were the main worries of urban older adults (61% and 60% respectively). Among rural residents, the areas where most were concerned were getting themselves infected or even their family members with the novel coronavirus (54%), prospectively living alone if family members were to be infected (53%), and being unable to purchase daily essentials (51%).

**Table 2:** Worries regarding the COVID-19 situation among older adults.

	Total (N = 1230)		Urban (%)	Rural (%)	*p*-value*
	n	%			
Getting myself or my family member infected with COVID-19	509	41.4	45.38	54.62	0.050
Personal and familial financial status	348	28.3	60.34	39.66	<0.001
Health status worsens because of missed medical appointments	220	17.9	54.55	45.45	<0.001
Accessibility to the treatment if infected with COVID-19	141	11.5	55.32	44.68	0.001
Unable to purchase essentials (i.e. food and medicine)	135	11.0	48.89	51.11	0.091
Living alone if any family member were to be infected with COVID-19	123	10.0	47.15	52.85	0.233
Conflict with my family while living together	54	4.4	61.11	38.89	0.004

Source: Impact of COVID-19 on Older Persons in Thailand Survey.

*Based on χ^2^ test comparing urban and rural residence.

Majority in the sample that had correct knowledge regarding COVID-19 were from the rural areas (Table 3). Based on the knowledge index, 43% of the sample knew the proper information based on the four items of COVID-19 to which 69% were from the rural areas. Many among the individuals in the survey were informed about the higher risks among people with chronic conditions, the mode of transmission of the disease, and the utility of mask wearing and handwashing. The aspect where fewer had proper knowledge was the incubation period of COVID-19 infection (55%).

**Table 3:** Correct knowledge about COVID-19 by residential area.

	Total		Urban (%)	Rural (%)	*p*-value*
	n	%			
Older persons with chronic conditions are at higher risk of getting infected with COVID-19.	1184	96.3	40.7	59.3	<0.001
COVID-19 can spread through a sneeze, a cough or even talking.	1210	98.4	41.7	58.3	0.037
Because the incubation period is 3–7 days, those who are exposed to COVID-19 infected cases should be quarantined for 7 days.	671	54.6	49.5	50.5	<0.001
Wearing a facemask and washing hands frequently can prevent the COVID-19 infection.	1219	99.1	41.9	58.1	0.146
**COVID-19 knowledge index**	530	43.1	30.6	69.4	<0.001

Source: Impact of COVID-19 on Older Persons in Thailand Survey.

*Based on χ^2^ test comparing urban and rural residence.

The preventive behaviour of the individuals sampled are presented in Table 4. Hand washing and mask wearing were most practiced with over 80% of the sample doing both. Around 77% follow physical distancing recommendations and about 62% were avoiding sharing meals to prevent the spread of the virus. Sheltering in place had the least adherence at 47% and many of those who followed this public health measure were from the rural areas. Overall, about 34% follow all five preventive behaviours and most of these individuals were rural residents (63%).

**Table 4:** Preventive behaviour regarding COVID-19 between urban and rural areas.

	Total		Urban (%)	Rural (%)	*p*-value*
	n	%			
Avoiding leaving the house	577	46.9	40.7	59.3	0.355
Following physical distancing	944	76.7	42.0	58.1	0.832
Washing hands frequently	1068	86.8	43.5	56.5	0.009
Wearing a facemask in public	1091	88.7	42.4	57.6	0.519
Avoiding meal sharing with others	758	61.6	38.8	61.2	0.003
**Preventive behaviour index**	413	33.6	37.3	62.7	0.015

Source: Impact of COVID-19 on Older Persons in Thailand Survey.

*Based on χ^2^ test comparing urban and rural residence.

In Table 5, the association of the sociodemographic and information source variables with knowledge and preventive behaviour regarding COVID-19 are shown. Regarding knowledge of COVID-19, rural residence was observed to have significant positive association, Higher education, being employed, and having the internet as their source of information were also observed to have positive relationship with proper knowledge regarding the virus. Being in the highest income category and having the television or radio as sources of information on the other hand had a significant negative association with the outcome.

**Table 5:** Logistic regression model of covariates with knowledge and preventive behaviour regarding COVID-19.

	Knowledge about COVID-19			Preventive behaviour		
	OR	95% CI	*p*-value	OR	95% CI	*p*-value
**Age groups** (Ref: 60–69)						
70–79	1.29	0.98–1.70	0.067	1.16	0.88–1.54	0.031
80+	1.41	0.95–2.11	0.090	1.96	1.32–2.90	<0.001
**Female**	1.16	0.91–1.49	0.237	1.04	0.80–1.33	0.791
**Rural residence**	2.65	2.07–3.40	<0.001	1.3	1.01–1.67	0.042
**Married**	1.09	0.82–1.44	0.553	1.44	1.07–1.92	0.054
**Living arrangement** (Ref: Living alone)						
Living with spouse only	0.92	0.48–1.77	0.806	1.24	0.63–2.41	0.532
Living with at least one child	0.82	0.47–1.40	0.460	0.93	0.53–1.64	0.805
Living with other people	1.03	0.57–1.87	0.918	0.79	0.42–1.47	0.456
**Education attainment** (Ref: Lower than primary level)						
Primary level [4–6 years	2.27	1.36–3.80	0.002	1.31	0.80–2.14	0.035
Higher than primary level	2.47	1.41–4.31	<0.001	1.66	0.97–2.84	0.045
**Employed during COVID-19 pandemic**	1.70	1.14–2.52	0.009	0.56	0.35–0.89	0.015
**Average annual income** (Ref:<10,000)						
10,000–29,999	0.90	0.66–1.24	0.518	0.83	0.60–1.14	0.248
≥30,000	0.79	0.56–1.02	0.048	0.68	0.50–0.93	0.015
**Owns radio/TV and received COVID-19 information through them**	0.55	0.31–0.96	0.034	0.68	0.39–1.17	0.166
**Owns mobile phone/computer and obtained information on COVID-19 through SMS or the internet**	1.93	1.47–2.55	<0.001	1.10	0.83–1.46	0.518
**Knowledge about COVID-19**	**-**	-	-	1.00	0.78–1.29	0.994
**Preventive behaviour**	1.03	0.78–1.29	0.983	-	-	-

Ref: Reference category, OR: Odds ratio, CI: Confidence interval.

In terms of preventive behaviour, it was observed that being in older age groups have positive association. This relationship was similar with rural residence and higher education attainment. Being employed during the pandemic and having an annual income of at least 30,000 Baht were found to have a negative relationship with preventive behaviours.

## Discussion

This study aimed to observe the level of knowledge about COVID-19 and the preventive actions among older adults of Thailand. The findings have also shown which among the sociodemographic characteristics of this vulnerable population and also their sources of information about the pandemic have associations with either health literacy factors. The related factors can then be considered whether they would be strengthened or remediated.

Most of the people in the sample were observed to have the knowledge about the higher risk of infection among people with chronic diseases, the mode of transmission, and the importance of handwashing and wearing a face mask. The level of awareness about the aforementioned aspects of COVID-19 were similarly observed in other LMIC settings [16, 18]. The difference from other studies though is that rural residents have a particularly higher level of awareness than those from urban areas.

Despite having challenges in accessing new media as the internet, Thailand has a community network for information dissemination that benefits the rural areas. In the late 1970s, the Village Health Volunteers (VHV) scheme was instituted as a key component of the country’s community-based public health system [21]. The VHV are responsible for raising health awareness and also monitoring the health status of particular districts. This community-based scheme had been shown to be critical in the rural communities toward addressing chronic diseases [22] and the surveillance of the Avian influenza [21].

Based on the observation in this study, the level of knowledge has a gap with preventive behaviour. In the case of the US [23], racial and other social disparities were identified to be limiting people’s capacities to perform protective actions against the disease. Socioeconomic status (SES) of adults was observed to contribute to the difficulties in adopting preventive behaviour as with the income-poor households in the Philippines [16] and Ecuador [24]. Although SES disparities were found in different societies, these are manifested differently depending on social settings.

In Thailand, the avoidance of leaving their houses was the least obliged preventive measure during the lockdown period. Employment during older ages has been noted to be prevalent in the country such that about a third in the said age group were working in the 1990s to the 2000s [25]. Employed older adults depend on their employment for income because most do not have access to the pension system [14]. Although concerns about being infected themselves during the pandemic was present among older adults, leaving their households and engaging in work was inevitable despite the higher risks that they have due to age.

Another observed aspect of the knowledge-behaviour gap was the sources of information. Obtaining information from traditional media which includes the radio and television was negatively associated with proper knowledge of COVID-19 while gathering information from novel media as the internet was positively related. Disparity in digital access and literacy has been observed among older adults [26]. In the present case, if older individuals were able to access laptops, smartphones and the like in their households, they can be better informed. As with having information from the radio or television media, it has been found that the extent of coverage may overwhelm people which subsequently leads to anxiety [8]. Being overwhelmed and becoming anxious with the situation can result to cognitive avoidance such that people act the way they do despite their awareness of risks`[27]. This is especially the case when health systems are weaker such as in LMICs because misinformation may arise and confound the understanding of people about the situation [16].

Employment is an indicator of socioeconomic status (SES) that was observed to have an opposing result toward knowledge and behaviour. But another aspect of SES is education. Higher levels of education attainment had been shown to be associated with better health statuses among Thai older persons [28, 29]. In the present study, it was also observed to be consistently associated with increased health literacy. The capability to evaluate various information and adopt the appropriate means to avoid a detrimental outcome are the benefits of having higher levels of education.

The final aspect of SES observed within this study was household income level and the result held much difference from education attainment. The negative relationship of higher income with knowledge and behaviour on COVID-19 is similar with the outcome found in Western China in terms of general health [30]. In the said study, the contention was people with higher income have greater access to health care and needs resulting to less motivation on doing protective actions or behaviour.

There are limitations to this study. Firstly, causation was not established here because of the cross-sectional design of the survey. A limited number of questions were also included in the survey. The items on constructing measures on knowledge and behaviour may not be comparable with other studies. The survey used also did not include attitudes toward COVID-19 which was a part of many studies relating knowledge and behaviour on preventing COVID-19 [15–18].

## Conclusion

This study observed the knowledge and preventive behaviour of a vulnerable population during the COVID-19 pandemic. Public health measures such as sheltering in place was viewed in many countries to be important in managing the transmission of the disease. This had various effects on older persons as they had higher risks on infection and mortality. It was important to consider the context of an LMIC because social and health disparities are present. This is the case with the loss of income during lockdown period in Thailand which was a hindrance to some older adults’ adherence to public health guidelines. The communication of health information has to consider the needs and lived experiences of various population subgroups. This is necessary to bridge the gap between knowledge and behaviour and work toward the well-being of vulnerable peoples including the older population during times of public health emergencies.
